# A Retrospective Study on the First Cerebrospinal Fluid Taken from External Ventricular Drainage Insertion in Meningitis Patients with Hydrocephalus

**DOI:** 10.21315/mjms2019.26.5.6

**Published:** 2019-11-04

**Authors:** Asma’ Mohamad Afifi, Jafri Malin Abdullah, Johari Adnan Siregar, Zamzuri Idris

**Affiliations:** 1 Department of Neurosciences, School of Medical Sciences, Universiti Sains Malaysia, Kubang Kerian, Kelantan, Malaysia; 2 Department of Neurosurgery, Hospital Sultanah Aminah Johor Bahru, Johor, Malaysia; 3 Department of Neurosurgery, Hospital Kuala Lumpur, Kuala Lumpur, Malaysia

**Keywords:** meningitis, hydrocephalus, external ventricular drainage

## Abstract

**Background:**

Ventriculoperitoneal (VP) shunting is a permanent form of cerebrospinal fluid (CSF) diversion that can be performed for hydrocephalus. Sterility of the CSF is an important prerequisite for permanent shunt placement. It has been hypothesised that in early stage of meningitis, ventricular CSF remains sterile. A study is conducted on the first CSF sample taken from patients suspected to have meningitic hydrocephalus.

**Method:**

A retrospective review case records of patients who had undergone external ventricular drainage (EVD) for suspected meningitic hydropcephalus in Hospital Sultanah Aminah Johor Bahru (HSAJB), Johor, Malaysia.

**Results:**

Fifty-one cases were analysed. Mean age of patients was 37.27 years old, with 64.7% of them was male. Univariate analysis revealed that the main parameters to determine CSF sterility were CSF glucose (95% CI, 0.852, 10.290, *P* = 0.001), CSF protein (CI 95%, 0.722, 14.898, *P* < 0.001), CSF gram stain (95% CI, 16.437, 0.877, *P* < 0.001 ) and CSF appearance ( 0.611, 6.362, *P* = 0.012). Multivariate analysis had proven that gram stain was the main parameter in the CSF analysis (CI 95%, 16.437, 0.029, *P* = 0.016). No significant differences in CSF results were observed from EVD and lumbar puncture.

**Conclusion:**

The most significant parameter in CSF to determine infection was gram stain.

## Introduction

Meningitis refers to the inflammation of the membranes (meninges) surrounding the brain and spinal cord (1). The incidence of bacterial meningitis varies globally and is about 1–2 cases per 100,000 people per year (2). It is most commonly caused by pneumococcus.

Clinically, meningitis is diagnosed using the classic triad of neck stiffness, fever, and altered consciousness. However, any two of these three symptoms are more common and are exhibited by up to 95% of meningitis patients (3). In addition, less than 50% of meningitis patients suffer from acute bacterial meningitis.

The pathogenesis of bacterial meningitis is not yet completely understood. However, there are four main pathogenic processes: colonisation, invasion into the bloodstream, survival in the bloodstream, and entry into the subarachnoid space. The subsequent damage occurs due to interaction between bacterial and host factors. Invasion of the bloodstream refers to both passing through or between the cells. Survival in the bloodstream requires evasion of the immune system. Meningitis, in the majority of patients, occurs following bacteraemia; however, the diagnosis of sinusitis and otitis media suggest direct spread to the central nervous system.

Examination of cerebrospinal fluid (CSF) is the gold standard among the diagnostic techniques employed for meningitis. High proportion of white blood cells (WBCs) indicates inflammation of the meninges; however, some meningitis patients exhibit normal level of WBCs. Such patients exhibit poor prognosis (4). Upon CSF analysis, the following factors might indicate the occurrence of meningitis: leukocyte count > 1000/mm^3^, ratio of CSF glucose levels to blood glucose levels < 0.23, protein level > 80 mg/dL, presence of Gram-positive bacteria, and other clinical manifestations that are compatible with meningitis (5). The confirmatory test for meningitis involves a blood culture.

There are several complications related to meningitis such as hydrocephalus, subdural collection, infarction cerebritis, ventriculitis, and brain abscess (6).

Hydrocephalus is most likely associated with an increased fatality. Radiological assessment for hydrocephalus includes ventricular enlargement with CSF seepage, effacement of sulci and gyri, ballooning of third ventricle, temporal horn dilatation > 3 mm, and bicaudate index (BCI). BCI is the width of the frontal horn at the level of caudate nuclei and foramen of Monroe divided by the corresponding diameter of the brain. Hydrocephalus is diagnosed when relative BCI index is more than 95% percentile of age.

Ventriculitis refers to the inflammation of the ventricles in the brain, and it is one of the complications of meningitis. The ventricles are responsible for the circulation of CSF throughout the brain. Ventriculitis is caused by infection in the ventricles, leading to swelling and inflammation (7).

When a patient is diagnosed with both meningitis and hydrocephalus, CSF diversion needs to be performed to maintain normal pressure in the brain. The best approach for CSF diversion is the external ventricular drainage (EVD) (8). EVD is frequently used to drain CSF, obtain a CSF sample, and manage brain CSF based on the total drainage status. For these patients, the risk of CSF infection caused due to a contaminated ventricular catheter ranges from 3.5%–21.9%. An early detection of a catheter-related ventriculitis is important for successful treatment which includes the removal or exchange of the catheter. The results from the bacteriological cultures, however, are not available before at least 48 h. Therefore, they cannot help in the early detection of incipient catheter contamination and therapeutic measures. Nosocomial ventriculitis is a potentially fatal condition which might contribute to a permanent adverse outcome on the patient (9). Besides that, infection can also be caused due to mismanagement during EVD due to inadequate training of the staff or caregivers.

In this study, the first CSF sample taken from EVD was analysed, from patients who diagnosed with meningitis along with hydrocephalus. The aim of this study was to determine the CSF status of first EVD sample after the diagnosis of the disease. To prove the hypothesis that the CSF sample from ventricle was sterile, it was also noteworthy to know how many patients eventually ended up with internalisation.

## Methods

A retrospective study was conducted on 51 patients who had undergone EVD insertions for treatment of obstructive hydrocephalus due to meningitis in Hospital Sultanah Aminah Johor Bahru (HSAJB), Johor, Malaysia during the period of January 2012 to December 2016. Convenient sampling method was employed in this study. The inclusion criteria included all meningitis patients who underwent EVD insertion and from whom the CSF sample was obtained from the surgery site. Meanwhile, the exclusion criteria included patients with ventriculitis, intracranial abscess, and other space occupying lesions, and from whom the CSF samples were obtained from lumbar puncture. Meningitis was diagnosed clinically based on clinical triad: headache, neck stiffness, and reduced consciousness. The radiological criteria were signified by the roundedness of the anterior horn, the ballooning of the third ventricle, the effacement of sulci and gyri, and lucent periventricular without meningeal enhancement (10). The primary endpoint of this study was to evaluate the first CSF sample obtained during the EVD insertion. The favourable outcome was defined as sterile CSF. An unfavourable outcome was defined as infection in the CSF based on the results of CSF culture. Secondary endpoint of this study was to analyse the parameters related to the infected CSF. Tertiary endpoint was to determine the possibility of employing VP shunt among the patients with sterile CSF.

Sex, CSF appearance, CSF protein, CSF glucose, CSF Gram stain status, WBC proportion and status of patient (either shunted or not) were collected to describe the variability of the study population. Data entry and analysis were done using SPSS programme for Windows version 22.0. The demography is presented in tabular form using the mean and standard deviation (SD) for numerical variables and numbers and percentages for categorical variables. Outcome predictors were analysed using simple and multiple logistic regression analyses so as to provide an odd ratio. The statistical significance was defined at *P* < 0.05. Comparison between CSF status and number of shunted patients is presented in Table 3. Association among meningitis and CSF status and need for shunt was assessed using simple logistic regression.

### Limitations

The data was obtained from the medical reports of a centre and we faced some difficulties in its assessment, especially when the patient was discharged from tertiary centre and subsequently underwent medical treatment in other centres.

## Results

Data was obtained from 51 meningitis patients that underwent EVD surgery at HSAJB, Johor, Malaysia during 2012 to 2017. The mean age of the patients was 37.27 years. Ten patients suffered from hydrocephalus as indicated by tumour and abscess and five patients exhibited ventriculitis as seen by CT contrast. Four patients were of young age (paediatric age group) and were, thus, were excluded.

The outcome cross tabulation is depicted in Table 1. In this table, there were two groups, one with favourable outcome and the other with unfavourable outcome. Favourable outcome refers to the parameters analysed in sterile CSF, while the unfavourable outcome refers to the parameters analysed in infected CSF. It was found that 41.17% of male patients and 17.64% of female patients had sterile CSF, while 23.5% of male and 17.64% of female patients had infected CSF. Among the patients with sterile CSF, 41.17% patients had clear CSF and only 13.72% patients had turbid CSF. Among the same subset of patients, the CSF of 54.90% patients harboured normal CSF protein levels, while the CSF of 3.92% patients exhibited high CSF protein levels. In addition, 54.90% patients with clear CSF exhibited normal glucose level and only 3.92% patients exhibited abnormal glucose level. CSF level of WBC was normal in 16 cases (31.37%) with sterile CSF, while 14 cases (27.5%) had low level of CSF WBC. Twenty-three patients (45.09%) with sterile CSF were shunted, whereas only seven patients (13.72%) were not shunted. CSF Gram stain was negative for more than 50% of cases and only 3.97% patients with clear CSF exhibited positive Gram staining. Hence, the overall unfavourable outcome was lesser than favourable outcome.

In Table 2, the association between four major parameters of CSF and CSF culture is presented, which were CSF protein, CSF glucose, CSF appearance and CSF Gram staining status. All these four parameters were found to be significantly associated with CSF culture. Then, multivariate logistic regression analysis was done. It was found that the CSF Gram staining status was the most significant parameter when compared to other parameters (CI 95%: 0.029, 16.437, *P* < 0.005). Thus, CSF Gram staining status was the most important parameter associated with CSF culture.

Table 3 shows the comparative data of VP shunt based on the CSF status. It was found that 45.1% patients with sterile CSF were shunted, and only 21.5% patients exhibited infected CSF and could not be shunted.

Table 4 shows the association between the CSF status and the VP shunt. A simple logistic regression test revealed that there was a significant association between CSF sample and VP shunt (CI 95%: 0.606, 1.285).

Table 5 shows the results of statistical analysis of first CSF sample obtained from ventricle and lumbar puncture, and essentially, they were similar to those obtained for the CSF finding of EVD and lumbar puncture.

## Discussion

Meningitis is characterised by fever, headache and meningismus with inflammation in the subarachnoid space as revealed by CSF pleocytosis. The time to presentation (acute, subacute, or chronic) and degree of illness differ based on the aetiology and appropriate initial management and treatment. Acute meningitis manifests within a few hours to several days. It is often infectious with a bacterial or viral causal factor, along with non-infectious aetiologies as revealed in the differential diagnoses (11).

In this study, only the first CSF samples obtained during EVD were analysed. Meningitis was diagnosed by evaluating the presence of clinical symptoms of meningitis, namely headache, fever and reduced consciousness level. CT scan of the brain was done, and hydrocephalus that needed intervention was noted. The EVD was performed to release the intracranial pressure due to hydrocephalus, as well as for CSF sampling. The first CSF sample taken during EVD was then analysed. Males exhibited higher tendency of developing meningitis along with hydrocephalus, as they were sexually active, busy working, and travelling, which can make them more susceptible to infection (12). Male patients exhibited more features of meningitis compared to female patients, with regards to clinical manifestations (13). Males often presented with high grade fever, and reduced consciousness level, along with occasional seizures (13). In children with suspected bacterial meningitis, showed sensitivities of 51% for neck stiffness and 53% for Kernig’s sign. In adults, this clinical finding have low accuracy, and cannot be excluded the possibility of bacterial meningitis (14).

The patients were categorised into two groups based on CSF appearance: clear CSF and non-clear CSF. A turbid CSF indicates higher levels of protein, white and red blood cells (>200 WBC per mm^3^ or > 400 RBC per mm^3^) and/or bacteria, and therefore, might suggest bacterial meningitis (18). Xanthochromia caused by red blood cell breakdown, one of it is because necrotic and haemorrhagic cerebral lesion common in HSV-1 encephalitis (15). In this study, 41.17% patients exhibited clear sterile CSF and 13.72% patients had non-clear sterile CSF. The appearance of CSF could only help in predicting whether CSF is sterile or not; it does not help in diagnosis (16).

Thirty-seven patients (72.5%) had normal levels CSF protein. High CSF protein level (>80 mg/dL) is one of the predictors of bacterial infection (17). However, in this study, the protein level was relatively normal due to the onset of the patient’s illness. Patients mainly came to emergency department after 1–2 days of fever, with reduced consciousness and headache. Some of these patients were also under antibiotic treatment for other diseases, which would also be suitable for the pathogen that caused meningitis.

CSF culture is the main determinant of the future management approach for these patients. Based on the study, CSF was sterile in 30 patients (58.8%). Some patients came after few days of infection, from district hospitals, after completion of a course of antibiotics. Further investigation showed that their CSF was sterile. The most common pathogen were *Streptococcus pneumonia* and *Hemophilus influenza* (18). This result suggested that not all meningitis patients suffered from ventriculitis or CSF infection (7).

Normal CSF glucose is 60%–70% of the patient’s serum glucose concentration. CSF glucose levels are used to discriminate bacterial meningitis from viral meningitis (12). Patients with bacterial meningitis usually have low levels of CSF glucose because of glycolysis by both white cells and the pathogen and impaired CSF. Most of the patients in this study exhibited normal CSF glucose levels. However, according to River et al., CSF analysis was not a reliable method for differentiating antibiotic-induced meningitis from the partially-treated bacterial meningitis. Some of the patients had been treated for meningitis before they developed hydrocephalus (19). Majority of the patients with normal glucose level were in sterile CSF group. Therefore, we could not conclude that patient with meningitis had infected CSF.

The level of WBCs in the blood is another parameter that needs to be considered for meningitis diagnosis. High levels of WBCs indicated an inflammatory process in the blood, which could be due to infection. From this study, the proportions of patients having normal and abnormal levels of WBCs were comparable. If WBC level is high, further investigation must be carried out before shunting procedure is carried out (11). It should be noted that some of the patients in this study had nosocomial infection and other comorbids such as other systemic infections due to a different treatment, old age, hypertension, and completed antibiotic course for meningitis. Therefore, the level of WBCs was only evaluated when the patient underwent EVD and not prior to that. Hence, the WBC level was similar for all groups. However, the association between high levels of WBCs and meningitis still remains controversial (20).

Gram staining status of CSF was the most useful factor to determine whether the cause of meningitis was bacterial; 89% of bacterial meningitis patients exhibited a positive initial Gram stain. In this study, 54.9% patients had sterile CSF exhibiting negative Gram staining. Only 3.97% patients had sterile CSF with positive Gram staining and 29.4% patients had infected CSF with negative Gram staining. Gram staining differentiates bacteria by the chemical and physical properties. Only the bacteria having peptidoglycan in their cell walls are presented as Gram-positive bacteria. In early acute meningitis, it is important to know which microorganism has infected CSF so as to devise a management approach accordingly (2). This result showed that majority of the patients had sterile CSF, based on Gram stain status, which could be confirmed with CSF culture.

In this study, less than 45.09% patients had sterile CSF and underwent VP shunt. VP shunt is a permanent diversion CSF that can be done only in patients with sterile CSF. In our study, 21.56% patients could not be shunted due to infected CSF. However, we did not evaluate the duration between the time of diagnosis and the time when shunt was inserted and which antibiotics were being administered to that patient.

The univariate logistic regression analysis was done to analyse four CSF criteria that were important in assessing CSF culture; these criteria include CSF protein, CSF Gram stain status, CSF glucose, and CSF appearance. The CSF was found to be cloudy, which may be due to the presence of high levels of WBCs, RBCs, bacteria and protein (21, 22). The CSF glucose ratio of less than 0.4 was more than 80% sensitive and 98% specific for diagnosis of bacterial meningitis in children of more than two months of age (23, 24). The CSF protein level is an important tool as it was elevated in virtually all patients with bacterial meningitis. These parameters can also help in the determination of the type of meningitis. However, it was not the most sensitive tool for identification of the type of infection (25). All the parameters with *P*-value < 0.05 were important. Therefore, we conducted another analysis to identify the best tool for diagnosis of infected CSF.

As shown in Table 2, four variables, which were the CSF glucose levels, the CSF protein levels, the CSF Gram staining level and the CSF appearance, were compared using the multivariate logistic regression analysis. The multivariate logistic regression analysis could determine the best parameter to indicate CSF infection. The results showed that CSF Gram staining status was the most significant parameter compared to others (*P* < 0.001) in indicating CSF infection. Hyponatremia, combined with a negative Gram stain, was highly suggestive of tuberculous meningitis. However, in this study, the natrium level was not analysed. The inflammation and the protein level obtained from pathogen was believed to be the cause of increased protein levels in the CSF and the inflammatory processes. The results of CSF culture were positive in 70%–85% patients who had not received any prior antimicrobial therapy. Therefore, since most of the patients had a negative Gram staining result and a clear CSF, the majority of patients with meningitis had sterile CSF.

In this study, the CSF culture status was also analysed based on the first CSF sample, and its association with the VP shunt was obtained. Twenty-three out of 51 (43.5 %) patients with sterile CSF ended up with shunts. Ten patients with infected CSF were shunted, mainly after completion of the antibiotics course. The rest of the patients were not shunted. Seven patients with clear CSF were not shunted because of multiple comorbidities, such as fragility and sepsis, and patients getting better with good medical management. Most of the remaining patients with infected CSF and those that were not shunted died due to medical problems. From this table, no conclusion could be derived.

The association between CSF culture and VP shunt was also analysed. From the univariate logistic regression study, the outcome was positive, showing a *P*-value of 0.039, showing significant association between CSF culture and VP shunt. Shunting only can be done on patient with sterile CSF.

Table 5 shows the results of analysis of the first CSF sample obtained from ventricle and lumbar puncture, which were comparable to the results of analysis of CSF obtained from EVD and lumbar puncture.

## Conclusion

Analysis of the first CSF sample could help in devising of better approach for management of meningitis patients. Based on this study, high protein levels (> 0.8) showed that the CSF was not sterile. The most important parameter to determine CSF status was the CSF gram staining status. Most patients exhibited normal parameters, and more than 60% of the patients had sterile CSF. Half of the patients ended up with shunting, which showed that most of them were diagnosed with meningitis, requiring a permanent diversion of CSF. However, it was not possible to confirm that the first sample of CSF obtained from a meningitis patient was sterile.

## Figures and Tables

**Table 1:** Descriptive statistic of first CSF sample analysis from EVD

Variable	CSF sterile *n* (%)	CSF non-sterile *n* (%)
**Sex**		
Male	21 (41.2)	12 (23.5)
Female	9 (17.6)	9 (17.6)
**CSF appearance**		
Clear	21 (14.2)	9 (17.6)
Non-clear	7 (13.7)	14 (27.5)
**CSF protein**		
Normal	28 (54.9)	9 (17.6)
High	2 (3.9)	12 (23.5)
**CSF glucose**		
Normal	28 (54.9)	10 (19.6)
High	2 (3.9)	11 (21.6)
**White blood cell**		
Normal	16 (31.4)	11 (21.6)
Low	14 (27.5)	10 (19.6)
**VP shunt**		
Patient shunted	23 (45.1)	10 (19.6)
Patient non-shunted	7 (13.7)	11 (21.6)
**CSF gram stains**		
Negative (no growth)	28 (54.9)	6 (11.8)
Positive	2 (4.0)	15 (29.4)

**Table 2:** Association between CSF culture and CSF biochemistry

Variable	Simple logistic regression			Multiple logistic regression		
	Wald statistic	Crude OR^a^ (95% CI^b^)	*P*-value	Wald statistic	Adj OR^d^ (95% CI^b^)	*P*-value c
**CSF protein**						
High		1				
Low	14.898	0.722 (0.15–0.254)	< 0.001			
**CSF glucose**						
Abnormal		1				
Normal	10.290	0.852 (0.012–0.345)	0.001			
**CSF appearance**						
Non-clear		1				
Clear	6.362	0.611 (0.065–0.709)	0.012			
**CSF gram stain**						
Positive		1			1	
Negative	16.437	0.877 (0.005–0.159)	< 0.001	16.437	0.029 (0.005–0.159)	< 0.001

a crude odds ratio,

b confidence interval,

c adjusted for CSF gram stain,

d adjusted odds ratio

**Table 3:** Comparison data of VP shunt based on CSF status

Variable	Patient with shunt *n* (%)	Patient without shunt *n* (%)	*P*-value^a^
**CSF status**			
Sterile	23 (45.1)	7 (13.7)	0.33
Non-sterile	10 (19.6)	11 (21.5)	

a adjusted for CSF status and VP shunt

**Table 4:** Association between CSF status and VP shunt

Variable	Wald statistic	Crude OR^a^ (95% CI^b^)	*P*-value^c^
VP shunt			
Not-shunted		1	
Shunted	4.376	0.606 (0.83–0.922)	0.036

a crude odds ratio,

b confidence interval,

c adjusted for CSF status and VP shunt

**Table 5:** Descriptive statistic of first CSF sample from ventricle and lumbar puncture

Variable	CSF ventricle *n* (%)	CSF lumbar puncture *n* (%)
**Sex**		
Male	5 (100)	5 (100)
**CSF appearance**		
Clear	4 (80)	5 (100)
Non-clear	1 (20)	0
**CSF protein**		
Normal	5 (100)	4 (80)
High	0	1 (20)
**CSF glucose**		
Normal	5 (100)	5 (100)
High	0	0
**CSF gram stains**		
Negative (no growth)	4 (80)	5 (100)
Positive (growth)	1	0

## References

[1] Greenberg SM (2016). Handbook of neurosurgery.

[2] McGill F, Heyderman RS, Panagiotou S, Tunkel AR, Solomon T (2016). Acute bacterial meningitis in adults. Lancet.

[3] Dharmarajan L, Salazar L, Hasbun R (2016). Gender differences in community-acquired meningitis in adults: clinical presentations and prognostic factors. J Meningitis.

[4] Friedmann PD, Samore MH (1998). Diagnostic characteristics of cerebrospinal fluid analysis for secondary meningitis in HIV-infected adults. J Investig Med.

[5] Kasanmoentalib ES, Brouwer MC, van der Ende A, van de Beek D (2010). Hydrocephalus in adults with community-acquired bacterial meningitis. Neurology.

[6] Wu H-M, Huang W-Y, Lee M-L, Yang AD, Chaou K-P, Hsieh L-Y (2011). Clinical features, acute complications, and outcome of *Salmonella meningitis* in children under one year of age in Taiwan. BMC Infect Dis.

[7] Shah GS (2018). Pyogenic ventriculitis and meningitis caused by *Streptococcus acidominimus* in humans: a case report. Am J Case Rep.

[8] Kirmani AR, Sarmast AH, Bhat AR (2015). Role of external ventricular drainage in the management of intraventricular haemorrhage: its complications and management. Surg Neurol Int.

[9] Pfisterer W, Mühlbauer M, Czech T, Reinprecht A (2003). Early diagnosis of external ventricular drainage infection: results of a prospective study. J Neurol Neurosurg Psychiatry.

[10] Pfister HW, Feiden W, Einhäupl KM (1993). Spectrum of complications during bacterial meningitis in adults. Results of a prospective clinical study. Arch Neurol.

[11] Bartt R (2012). Acute bacterial and viral meningitis. Continuum (Minneap Minn).

[12] Pfister H, Feiden W, Einhäupl K (1993). Spectrum of complications during bacterial meningitis in adults: results of a prospective clinical study. Arch Neurol.

[13] Van de Beek D, de Gans J, Tunkel AR, Wijdicks EFM (2006). Community-acquired bacterial meningitis in adults. N Engl J Med.

[14] Brouwer MC, Thwaites GE, Tunkel AR, van de Beek D (2012). Dilemmas in the diagnosis of acute community-acquired bacterial meningitis. Lancet.

[15] Machado Ldos R, Livramento JA, Vianna LS (2013). Cerebrospinal fluid analysis in infectious diseases of the nervous system: when to ask, what to ask, what to expect. Arq Neuropsiquiatr.

[16] Venkatesh B, Scott P, Ziegenfuss M (2000). Cerebrospinal fluid in critical illness. Crit Care Resusc.

[17] Tunkel AR, Hartman BJ, Kaplan SL, Kaufman BA, Roos KL, Scheld WM (2004). Practice guidelines for the management of bacterial meningitis. Clin Infect Dis.

[18] Durand ML, Calderwood SB, Weber DJ, Miller SI, Southwick FS, Caviness VSJ (1993). Acute bacterial meningitis in adults: a review of 493 episodes. N Engl J Med.

[19] Nigrovic LE, Kimia AA, Shah SS, Neuman MI (2012). Relationship between cerebrospinal fluid glucose and serum glucose. N Engl J Med.

[20] Lembo MR, Rubin HD, Krowchuk D, Mccarthy LP (1991). Peripheral white blood cell counts and bacterial meningitis: implications regarding diagnostic efficacy in febrile children.

[21] Bekairy AMA, Harbi SA, Alkatheri AM, Dekhail SA, Swaidan LA, Khalidi N (2014). Bacterial meningitis: an update review. Afr J Pharm Pharmacol.

[22] Hoogman M, van de Beek D, Weisfelt M, de Gans J, Schmand B (2007). Cognitive outcome in adults after bacterial meningitis. J Neurol Neurosurg Psychiatry.

[23] Ginsberg L (2004). Difficult and recurrent meningitis. J Neurol Neurosurg Psychiatry.

[24] Hoffman O, Weber JR (2009). Pathophysiology and treatment of bacterial meningitis. Ther Adv Neurol Disord.

[25] Sakushima K, Hayashino Y, Kawaguchi T, Jackson JL, Fukuhara S (2011). Diagnostic accuracy of cerebrospinal fluid lactate for differentiating bacterial meningitis from aseptic meningitis: a meta-analysis. J Infect.

